# Genetic Evidence Supporting the Causal Role of Homocysteine in Chronic Kidney Disease: A Mendelian Randomization Study

**DOI:** 10.3389/fnut.2022.843534

**Published:** 2022-04-14

**Authors:** Yang Xiong, Yangchang Zhang, Fuxun Zhang, Changjing Wu, Peiyi Luo, Feng Qin, Jiuhong Yuan

**Affiliations:** ^1^Andrology Laboratory, West China Hospital, Sichuan University, Chengdu, China; ^2^Department of Urology, West China Hospital, Sichuan University, Chengdu, China; ^3^Department of Epidemiology and Health Statistics, School of Public Health and Management, Chongqing Medical University, Chongqing, China; ^4^Department of Nephrology, Kidney Research Institute, West China Hospital, Sichuan University, Chengdu, China

**Keywords:** chronic kidney disease, causal estimates, estimated glomerular filtration rate, homocysteine, Mendelian randomization

## Abstract

**Background:**

The causal relationship between homocysteine (Hcy) levels and chronic kidney disease (CKD) remains unclear. This study was performed to estimate the potential causal effects of Hcy on the estimated glomerular filtration rate (eGFR) and CKD.

**Materials and Methods:**

The single nucleotide polymorphisms (SNPs) associated with one standard deviation (SD) Hcy increase were identified using the genome-wide association study (GWAS). The summary statistics of the eGFR and CKD were from the CKDGen project in the European ancestry and the Population Architecture using Genomics and Epidemiology (PAGE) project in the non-European ancestry. Two-sample Mendelian randomization (MR) analyses were used in this study to verify the causal effects among Hcy, eGFR, and CKD.

**Results:**

The results showed that 1-SD Hcy increase was causally associated with eGFR decline in the CKDGen project (β = −0.027 log ml.min^–1^/1.73 m^2^, *p* < 0.01 for the overall cohort; β = −0.028 log ml.min^–1^/1.73 m^2^, *p* < 0.01 after excluding the patients with diabetes). In addition, 1-SD Hcy increase was associated with a 1.32-fold risk of CKD in the PAGE project (95% CI = 1.06–1.64, *p* < 0.05). The association was directionally similar in the CKDGen project [odds ratio (OR) = 1.08, 95% CI = 0.97–1.44, *p* = 0.098]. The pooled OR of CKD was 1.24 (95% CI = 1.07–1.44, *p* < 0.05) per 1-SD Hcy increase.

**Conclusion:**

Using genetic data, Hcy increase is causally associated with renal function injury and further CKD.

## Introduction

Currently, chronic kidney disease (CKD) is prevalent globally and places heavy adverse effects on populations. The diagnosis of CKD varies at different times and countries. However, as of now, the evidence of reduced kidney function revealed by the estimated glomerular filtration rate (eGFR) <60 ml.min^–1^/1.73 m^2^, or the biomarkers of kidney damage, lasting for ≥3 months, has been gradually accepted as a guideline (1). According to this standard, the prevalence of CKD is 10.8% in a national survey in China, 6.9% in the United States, 7.9% in Korea, and 10.0% in Switzerland (2–5). As indicated by the current epidemiologic data, the incidence of CKD remains stable; however, the high prevalence still requires requisite actions to attenuate the early onset of CKD (1).

Previous studies have reported heterogeneous risk factors, such as diabetes, metabolic syndrome, dyslipidemia, and primary kidney diseases (6). Among them, the role of metabolism in the onset of CKD has attracted the attention of researchers. The harms from risky metabolites, such as homocysteine (Hcy), are long-standing when exposed (7, 8). Hcy is a crucial molecule in transferring methyl. However, according to a cross-sectional report in 17,010 subjects from Cohen et al. (9), a higher Hcy concentration is associated with lower eGFR, and patients with CKD are found to have a higher concentration of Hcy than the non-CKD counterpart. Consistent findings are replicated in a longitudinal cohort in the middle-aged and older adults Chinese (10). A positive association between Hcy and CKD is disclosed; however, most studies are based on observational findings, which can not overcome the endogeneity and present unbiased estimates. Clear causal associations among Hcy, CKD, and eGFR, will aid the clinical practice but are still lacking.

Mendelian randomization (MR) is an epidemiological method based on genetic variants. By using genetic variables as instruments to replace exposures (i.e., Hcy) on outcomes (i.e., CKD and eGFR), this method exerts more substantial causal inference power on estimating causal links (11). Single nucleotide polymorphisms (SNPs) are assorted randomly during meiosis leading to a random distribution of genetic variants, which favors avoiding the reversed causation and confounding (12). The two-sample MR study between Hcy and CKD has not been performed to verify their causal association. In this study, a conventional MR method is used to estimate whether the increase of Hcy concentration is causally linked with the onset of CKD and eGFR decline.

## Materials and Methods

### Genetic Instrument Selection

The SNPs associated with total Hcy (tHcy) concentrations were identified in ten previous studies and then meta-analyzed by Van Meurs et al. (13). All the genetic instruments were associated with tHcy at the genome-wide significance threshold (*p* < 5 × 10^–8^). To confirm the independence of selected SNPs, linkage disequilibrium (LD) among SNPs for tHcy was assessed using a PLINK clumping method based on 1,000 Genomes European reference panel. SNPs with LD *r*^2^ < 0.01 at a 10 Kb window were considered independent SNPs and used as instrumental variables in this study. Finally, a total of 14 significant SNPs associated with tHcy were obtained from this meta-analysis. As indicated by Van Meurs et al. (13), the effects of obtained SNPs were scaled to one standard deviation (SD) increase of tHcy. A detailed description of the used genetic instruments is displayed in Supplementary Table 1.

### Genetic Summary Data of Chronic Kidney Disease and Estimated Glomerular Filtration Rate

The summary data of CKD and eGFR were obtained from two projects, the CKDGen consortium and the Population Architecture using Genomics and Epidemiology (PAGE) study (14, 15). The CKDGen consortium meta-analyzed the genome-wide association study (GWAS) data of CKD in 23 cohorts. These cohorts were performed in the European ancestry with a total of 480,698 participants (41,395 cases and 439,303 controls). The diagnosis of CKD was mainly based on the eGFR calculated by Schwartz formula (<18 years) and the CKD Epi-equation for adults (>18 years), respectively (16, 17). The cut-off was set as eGFR < 60 ml.min^–1^/1.73 m^2^ (17, 18). The summary data from the CKDGen consortium can be downloaded from the United Kingdom Medical Research Council Integrative Epidemiology Unit (MRC-IEU) Open GWAS database.

The PAGE study was performed in a non-European ancestry, such as Hispanic/Latino (*n* = 22,216), African American (*n* = 17,299), Asian (*n* = 4,680), Native Hawaiian (*n* = 3,940), Native American (*n* = 652), or other (*n* = 1,052) (15). CKD was defined as an eGFR (estimated by the CKD Epi-equation) < 60 ml.min^–1^/1.73 m^2^ (17, 18). Different from the CKDGen consortium, in the PAGE study, CKD patients with end-stage renal disease were excluded. Additionally, CKD was also adjusted for age, sex, race, study, study center, and comorbidities. The summary statistics were available through the GWAS Catalog database.

### Statistical Analyses

The random effect inverse-variance-weighted (IVW) method was used to test the causal association among tHcy, CKD, and eGFR. When assuming all the SNPs were valid, the IVW method combines the effects of individual SNPs and produces an overall weighted effect (19). The results of IVW were considered the main results due to the potential observed heterogeneity (20). The estimates from the CKDGen and PAGE by IVW were pooled using the fixed-effect meta-analysis method (21). Additionally, we adopted another four approaches for sensitivity analyses, which included MR-Egger, the weighed median, the simple mode, and the weighted mode.

The MR-Egger regression approach considers the effects of directional pleiotropy (22). An intercept term is introduced in the weighted regression model, and the directional pleiotropy is revealed when the intercept term is significantly away from zero in statistics. By combining multiple instruments into one single causal estimate, the weighted median approach can produce consistent estimates even when up to 50% of the genetic instruments are invalid (23). Proposed by Hartwig et al. (24), the mode-based estimate can consistently estimate the true causal effect when most instruments with consistent MR estimates are valid.

Moreover, the leave-one-out analysis was employed to verify the robustness of the casual estimation. Cochran’s *Q*-test was used to evaluate the heterogeneity between the genetic instruments. The value of *p* < 0.05 (two-sided) is considered as significant in statistics. All the analyses were finished using R software (version 3.6.5).

## Results

### Causal Association Between Total Homocysteine and Estimated Glomerular Filtration Rate With and Without Diabetes

**FIGURE 1:**
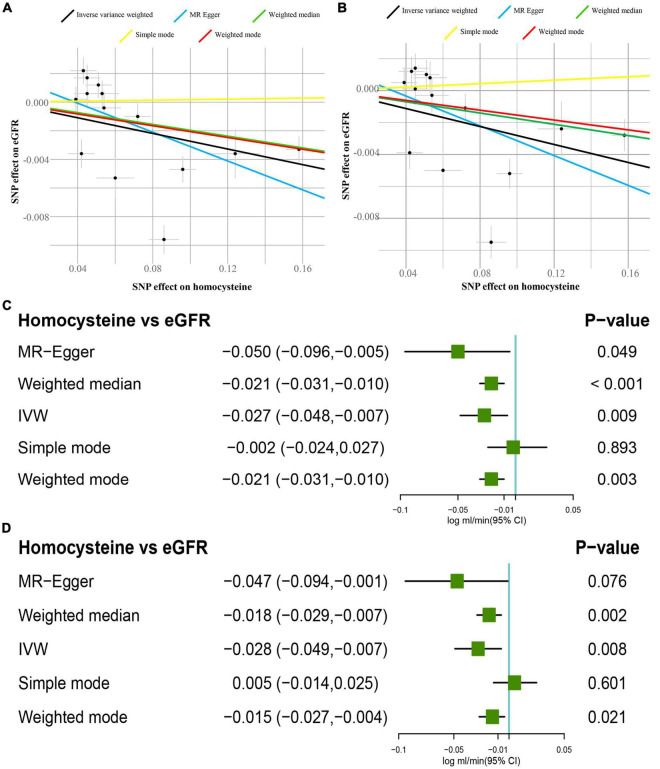
Causal estimates of 1-SD homocysteine (Hcy) increase on eGFR. **(A)** Scatter plot of the effect size of each SNP on tHcy and eGFR in the CKDGen project with diabetic patients. **(B)** Scatter plot of the effect size of each SNP on tHcy and eGFR in the CKDGen project excluding the patients with diabetes. **(C)** Results of the Mendelian randomization (MR) analyses estimating the causal association between 1-SD tHcy increase and eGFR in the CKDGen study with diabetic patients. **(D)** Results of the MR analyses estimating the causal association between 1-SD tHcy increase and eGFR in the CKDGen study excluding the patients with diabetes. “TwoSampleMR” package in R 3.6.5 was used to perform MR analyses. SD, standard deviation; tHcy, total homocysteine; IVW, inverse variance weighted method; eGFR, estimated glomerular filtration rate; SNP, single nucleotide polymorphism.

In Figure 1C, participants in the CKDGen study with diabetes were not excluded, and the IVW approach revealed that 1-SD increase of tHcy led to a decreased eGFR (β = −0.027 log ml.min^–1^/1.73 m^2^, *p* < 0.01). The decreasing trend of eGFR remained consistent in the results of MR-Egger, the weighted median, and the weighted mode method (*p* < 0.05). In Figure 1D, after excluding the patients with diabetes, the IVW approach disclosed same decreasing trend of eGFR (β = −0.028 log ml.min^–1^/1.73 m^2^, *p* < 0.01). In addition, this finding was replicated by the weighted median and weighted mode method (*p* < 0.05). The scatter plots of the SNPs-CKD association against SNPs-tHcy association in the CKDGen study with or without diabetic patients were shown in Figures 1A,B, respectively.

When not excluding the patients with diabetes, the intercept was 0.001 in the MR-Egger regression model (*p* = 0.28), indicating no pleiotropy for the genetic instruments. Similarly, after excluding the patients with diabetes, the intercept was 0.002 in the MR-Egger regression model (*p* = 0.41), showing no pleiotropy. In Supplementary Figures 1A,B, before and after excluding patients with diabetes, the leave-one-out analyses concurrently showed that leaving one single SNP out produced consistent findings as stated above. It demonstrated that no influential SNPs existed in the tHcy-eGFR causal association. The forest plots visualizing the estimates of each SNP on eGFR before and after excluding patients with diabetes are displayed in Supplementary Figures 2A,B, respectively. The Cochran’s *Q* statistic indicated the signs of heterogeneity before and after excluding patients with diabetes (before: *Q* value = 12 for the MR-Egger regression, *p* < 0.05; after: *Q* value = 13 for the IVW method, *p* < 0.05).

### Causal Association Between Total Homocysteine and Chronic Kidney Disease

**FIGURE 2:**
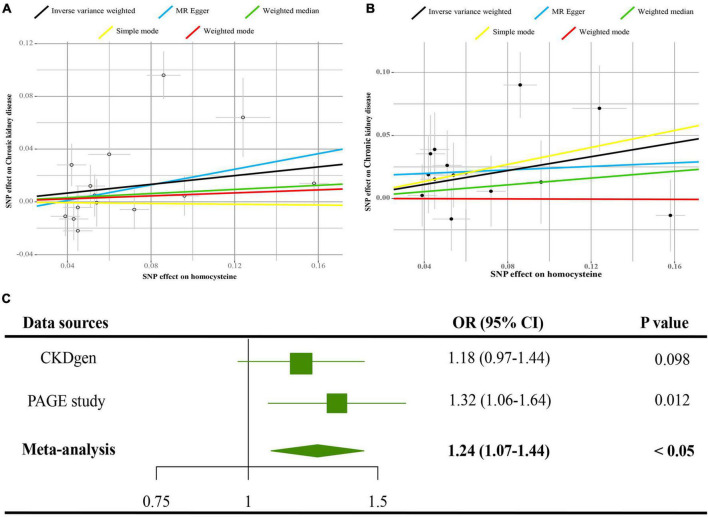
Causal estimates of 1-SD Hcy increase on CKD. **(A)** Scatter plot of the effect size of each SNP on tHcy and CKD in the CKDGen project. **(B)** Scatter plot of the effect size of each SNP on tHcy and CKD in the Population Architecture using Genomics and Epidemiology (PAGE) project. **(C)** Results of the MR analyses estimating the causal association between 1-SD tHcy increase and CKD in the CKDGen and PAGE project. SD, standard deviation; Hcy, homocysteine; IVW, inverse variance weighted method; SNP, single nucleotide polymorphism; CKD, chronic kidney disease.

In Figure 2C, data from PAGE study supported that 1-SD increase of tHcy increased the risks of CKD [odds ratio (OR) = 1.32, 95% CI = 1.06–1.64, *p* < 0.05]. Similarly, data from the CKDGen disclosed an increased OR (OR = 1.08, 95% CI = 0.97–1.44), but not significant in statistics (*p* = 0.098). The pooled estimates from CKDGen and PAGE by fixed-effect meta-analysis method revealed a significant causal association between tHcy and CKD (pooled OR = 1.24, 95% CI = 1.07–1.44, *p* < 0.05). The scatter plots of the SNPs-CKD association against SNPs-tHcy association in the CKDGen and PAGE study are displayed in Figures 2A,B, respectively.

The Egger intercepts in the MR-Egger regression were −0.01 (*p* = 0.54) and 0.02 (*p* = 0.38) in the CKDGen and PAGE study, respectively, indicating no directional pleiotropy. Cochran’s *Q*-test revealed no heterogeneity for the data from the PAGE study (*Q* value = 14.49 for the MR-Egger method, *p* = 0.27; *Q* value = 15.51 for the IVW method, *p* = 0.27). However, Cochran’s *Q* statistic indicated a sign of heterogeneity for the data from the CKDGen study (*Q* value = 12 for the MR-Egger regression, *p* < 0.05; *Q* value = 13 for the IVW method, *p* < 0.05). The leave-one-out analyses for the data from the CKDGen and PAGE are displayed in Supplementary Figures 3A,B, showing no influential SNPs in the association between tHcy and CKD. The estimates of each SNP on CKD are visualized as forest plots in Supplementary Figures 4A,B. In Supplementary Figure 5, the estimates in the sensitivity analyses by MR-Egger regression, the weighed median, the simple mode, and the weighted mode, reported an insignificant association between tHcy and CKD (*p* > 0.05), suggesting the limited power in detecting the causal relation.

## Discussion

In this two-sample MR study, the adverse effects of 1-SD tHcy increase are noted for the eGFR and CKD for the European and non-European ancestry. Consistent findings are replicated in participants with or without diabetes. These estimates provide novel causal evidence linking tHcy increase to renal function injury, which avoids biases from endogeneity.

As a pivotal role in the complex derangement of sulfur metabolism peculiar to patients with CKD, Hcy has been investigated in clinical and experimental settings frequently. The negative association between tHcy and CKD is documented in most but not all observational studies (25–27). In a cross-sectional survey enrolling 1,581 participants in China, subjects with increased serum Hcy were found to have a 5.76-fold risk of CKD than the counterparts with normal serum Hcy levels (25). Similarly, as reported by Levi et al., increased serum tHcy was associated with faster eGFR decline (26). Additionally, subjects with serum Hcy ≤15 μmol/L were observed to have a 4.85-fold risk of CKD than the normal tHcy participants during a 7.75 year median follow-up time (26).

However, in a retrospective cross-sectional study with multi-center data, a total of 22,043 adult Taiwanese were analyzed, which found that tHcy was associated with CKD in women but not in men after adjusted for seven covariates (27). In the baseline characteristics, the eGFR of men increased across the four groups (≤8.19 μmol/L, 8.20–9.84 μmol/L, 9.85–11.81 μmol/L, and ≥11.82 μmol/L), indicating a positive effect of tHcy on CKD. The residual confounding may lead to the discrepancy, which MR avoids. This study contributes to clarify the inconsistent findings.

The mechanisms linking tHcy to renal function injury may be mediated by insufficient autophagy. In a diet-induced hyper-Hcy rat model, increased serum Hcy could decrease the expression of a critical transcription factor of autophagy-related genes, the transcription factor EB (TFEB), which further inhibits the activation of TFEB-mediated autophagy (28). In previous studies, it is noted that the endothelial autophagy of kidneys is crucial for protecting glomeruli from oxidative stress and accordingly maintaining the integrity of glomerular capillaries (29). Therefore, therapies for reducing the serum tHcy can be considered in the high tHcy population, possibly lessening the incidence of renal function impairment and CKD.

This study has some merits and demerits. The major strength is the MR design, which enhances the power of causal inference of tHcy on eGFR and CKD. The adverse effects of 1-SD Hcy increase on eGFR decline remain consistent in participants with and without diabetes. The principal demerit of this study may be the possible pleiotropy, indicating that these genetic variants may be associated with confounding and then lead to the renal function injury, not *via* the tHcy. However, the insignificant results of the MR-Egger regression approach indicate that the bias may be minimal. Additionally, although the causal estimates of tHcy on CKD and tHcy on eGFR are significant in the PAGE and CKDGen project, respectively, the findings of tHcy on CKD in the CKDGen project are of marginal significance. The discrepancy may be accounted for the different ancestries in the PAGE and CKDGen projects.

To conclude, this MR study provides causal evidence supporting the adverse effects of tHcy on eGFR and CKD. Interventions to reduce Hcy, such as folic acid supplement, low-Hcy diet, and Hcy-lowering drugs, may be beneficial to protect renal function.

## Conclusion

Using genetic data, this study provides causal evidence that higher tHcy concentration may lead to renal function injury and further CKD.

## Data Availability Statement

Publicly available datasets were analyzed in this study. This data can be found here: https://gwas.mrcieu.ac.uk/.

## Ethics Statement

Ethical review and approval were waived for this study. Informed consent was obtained from all subjects in the original genome-wide association studies.

## Author Contributions

YX and YZ performed the data analyses and wrote the manuscript. CW, FZ, PL, and FQ revised the manuscript. JY participated in the study design and helped draft the manuscript. All authors contributed to the article and approved the submitted version.

## Conflict of Interest

The authors declare that the research was conducted in the absence of any commercial or financial relationships that could be construed as a potential conflict of interest.

## Publisher’s Note

All claims expressed in this article are solely those of the authors and do not necessarily represent those of their affiliated organizations, or those of the publisher, the editors and the reviewers. Any product that may be evaluated in this article, or claim that may be made by its manufacturer, is not guaranteed or endorsed by the publisher.

## Supplementary Material

Supplementary Figure 1 The leave-one-out sensitivity analyses between 1-SD Hcy increase and eGFR. **(A)** Results of the leave-one-out analyses in the CKDGen project with diabetic patients. **(B)** Results of the leave-one-out analyses in the CKDGen project excluding patients with diabetes. SD, standard deviation; Hcy, homocysteine; eGFR, estimated glomerular filtration rate.

Supplementary Figure 2 The results of Mendelian randomization (MR) analyses of causal associations between each Hcy SNP and eGFR. **(A)** Forest plot of the MR analyses in the CKDGen project with diabetic patients. **(B)** Forest plot of the MR analyses in the CKDGen project excluding patients with diabetes. Hcy, homocysteine; eGFR, estimated glomerular filtration rate; SNP, single nucleotide polymorphism.

Supplementary Figure 3 The leave-one-out sensitivity analyses between 1-SD Hcy increase and CKD. **(A)** Results of the leave-one-out analyses in the CKDGen project. **(B)** Results of the leave-one-out analyses in the Population Architecture using Genomics and Epidemiology (PAGE) project. SD, standard deviation; Hcy, homocysteine; CKD, chronic kidney disease.

Supplementary Figure 4 The results of MR analyses of causal associations between each Hcy SNP and CKD. **(A)** Forest plot of the MR analyses in the CKDGen project. **(B)** Forest plot of the MR analyses in the PAGE project. Hcy, homocysteine; CKD, chronic kidney disease; SNP, single nucleotide polymorphism.

Supplementary Figure 5 Causal estimates of 1-SD Hcy increase on CKD. **(A)** Results of the MR analyses estimating the causal association between 1-SD tHcy increase and CKD in the CKDGen project. **(B)** Results of the MR analyses estimating the causal association between 1-SD tHcy increase and CKD in the PAGE project. SD, standard deviation; Hcy, homocysteine; IVW, inverse variance weighted method; OR, odds ratio; CKD, chronic kidney disease.

Supplementary Table 1 Single nucleotide polymorphisms (SNPs) used as genetic instruments in this study. SNPs, single nucleotide polymorphisms.

## References

[1] Webster AC Nagler EV Morton RL Masson P. Chronic kidney disease. *Lancet.* (2017) 389:1238–52. 27887750 10.1016/S0140-6736(16)32064-5

[2] Zhang L Wang F Wang L Wang W Liu B Liu J Prevalence of chronic kidney disease in china: a cross-sectional survey. *Lancet.* (2012) 379:815–22. 22386035 10.1016/S0140-6736(12)60033-6

[3] Murphy D Mcculloch CE Lin F Banerjee T Bragg-Gresham JL Eberhardt MS Trends in prevalence of chronic kidney disease in the united states. *Ann Intern Med.* (2016) 165:473–81. 10.7326/M16-0273 27479614 PMC5552458

[4] Ji E Kim YS. Prevalence of chronic kidney disease defined by using ckd-epi equation and albumin-to-creatinine ratio in the korean adult population. *Korean J Intern Med.* (2016) 31:1120–30. 10.3904/kjim.2015.193 27017386 PMC5094925

[5] Ponte B Pruijm M Marques-Vidal P Martin PY Burnier M Paccaud F Determinants and burden of chronic kidney disease in the population-based colaus study: a cross-sectional analysis. *Nephrol Dial Transplant.* (2013) 28:2329–39. 10.1093/ndt/gft206 23825103

[6] Kronenberg F. Emerging risk factors and markers of chronic kidney disease progression. *Nat Rev Nephrol.* (2009) 5:677–89. 10.1038/nrneph.2009.173 19935815

[7] Karmin O Siow YL. Metabolic imbalance of homocysteine and hydrogen sulfide in kidney disease. *Curr Med Chem.* (2018) 25:367–77. 10.2174/0929867324666170509145240 28486919

[8] Ostrakhovitch EA Tabibzadeh S. Homocysteine in chronic kidney disease. *Adv Clin Chem.* (2015) 72:77–106. 10.1016/bs.acc.2015.07.002 26471081

[9] Cohen E Margalit I Shochat T Goldberg E Krause I. The relationship between the concentration of plasma homocysteine and chronic kidney disease: a cross sectional study of a large cohort. *J Nephrol.* (2019) 32:783–9. 10.1007/s40620-019-00618-x 31165981

[10] Kong X Ma X Zhang C Su H Xu D. Hyperhomocysteinemia increases the risk of chronic kidney disease in a chinese middle-aged and elderly population-based cohort. *Int Urol Nephrol.* (2017) 49:661–7. 10.1007/s11255-016-1452-3 27822673

[11] Smith GD Ebrahim S. ‘Mendelian randomization’: can genetic epidemiology contribute to understanding environmental determinants of disease?. *Int J Epidemiol.* (2003) 32:1–22. 10.1093/ije/dyg070 12689998

[12] Emdin CA Khera AV Kathiresan S. Mendelian randomization. *JAMA.* (2017) 318:1925–6. 29164242 10.1001/jama.2017.17219

[13] Van Meurs JB Pare G Schwartz SM Hazra A Tanaka T Vermeulen SH Common genetic loci influencing plasma homocysteine concentrations and their effect on risk of coronary artery disease. *Am J Clin Nutr.* (2013) 98:668–76. 10.3945/ajcn.112.044545 23824729 PMC4321227

[14] Pattaro C Teumer A. Genetic associations at 53 loci highlight cell types and biological pathways relevant for kidney function. *Nat Commun.* (2016) 7:10023. 10.1038/ncomms10023 26831199 PMC4735748

[15] Wojcik GL Graff M Nishimura KK Tao R Haessler J Gignoux CR Genetic analyses of diverse populations improves discovery for complex traits. *Nature.* (2019) 570:514–8. 10.1038/s41586-019-1310-4 31217584 PMC6785182

[16] Schwartz GJ Schneider MF Maier PS Moxey-Mims M Dharnidharka VR Warady BA Improved equations estimating gfr in children with chronic kidney disease using an immunonephelometric determination of cystatin c. *Kidney Int.* (2012) 82:445–53. 10.1038/ki.2012.169 22622496 PMC3433576

[17] Inker LA Schmid CH Tighiouart H Eckfeldt JH Feldman HI Greene T Estimating glomerular filtration rate from serum creatinine and cystatin c. *N Engl J Med.* (2012) 367:20–9. 22762315 10.1056/NEJMoa1114248 PMC4398023

[18] Xiong Y Zhang F Wu C Zhang Y Huang X Qin F The circadian syndrome predicts lower urinary tract symptoms suggestive of benign prostatic hyperplasia better than metabolic syndrome in aging males: a 4-year follow-up study. *Front Med.* (2021) 8:715830. 10.3389/fmed.2021.715830 34621761 PMC8490706

[19] Burgess S Butterworth A Thompson SG. Mendelian randomization analysis with multiple genetic variants using summarized data. *Genet Epidemiol.* (2013) 37:658–65. 10.1002/gepi.21758 24114802 PMC4377079

[20] Burgess S Thompson SG. *Mendelian Randomization: Methods for Using Genetic Variants in 494 Causal Estimation.* Boca Raton, FL: CRC Press (2015). p. 495

[21] Yuan S Larsson SC. Coffee and caffeine consumption and risk of kidney stones: a mendelian randomization study. *Am J Kidney Dis.* (2022) 79:9–14.e1. 10.1053/j.ajkd.2021.04.018 34690004

[22] Bowden J Davey Smith G Burgess S. Mendelian randomization with invalid instruments: effect estimation and bias detection through egger regression. *Int J Epidemiol.* (2015) 44:512–25. 10.1093/ije/dyv080 26050253 PMC4469799

[23] Bowden J Davey Smith G Haycock PC Burgess S. Consistent estimation in mendelian randomization with some invalid instruments using a weighted median estimator. *Genet Epidemiol.* (2016) 40:304–14. 27061298 10.1002/gepi.21965 PMC4849733

[24] Hartwig FP Davey Smith G Bowden J. Robust inference in summary data mendelian randomization via the zero modal pleiotropy assumption. *Int J Epidemiol.* (2017) 46:1985–98. 10.1093/ije/dyx102 29040600 PMC5837715

[25] Chao MC Hu SL Hsu HS Davidson LE Lin CH Li CI Serum homocysteine level is positively associated with chronic kidney disease in a taiwan chinese population. *J Nephrol.* (2014) 27:299–305. 10.1007/s40620-013-0037-9 24430766

[26] Levi A Cohen E Levi M Goldberg E Garty M Krause I. Elevated serum homocysteine is a predictor of accelerated decline in renal function and chronic kidney disease: a historical prospective study. *Eur J Intern Med.* (2014) 25:951–5. 10.1016/j.ejim.2014.10.014 25457436

[27] Chuang CH Lee YY Sheu BF Hsiao CT Loke SS Chen JC Homocysteine and c-reactive protein as useful surrogate markers for evaluating ckd risk in adults. *Kidney Blood Press Res.* (2013) 37:402–13. 10.1159/000355722 24247268

[28] Zhang S Zhang Y Zhang X Luo C Cao Y Ji D Nitrative stress-related autophagic insufficiency participates in hyperhomocysteinemia-induced renal aging. *Oxid Med Cell Longev.* (2020) 2020:4252047. 10.1155/2020/4252047 32047576 PMC7007752

[29] Tang C Livingston MJ Liu Z Dong Z. Autophagy in kidney homeostasis and disease. *Nat Rev Nephrol.* (2020) 16:489–508. 10.1038/s41581-020-0309-2 32704047 PMC7868042

