# Supplementary figures and images for: Genetic Evidence Supporting the Causal Role of Homocysteine in Chronic Kidney Disease: A Mendelian Randomization Study
Source: Front Nutr. 2022 Apr 14;9:843534. doi: 10.3389/fnut.2022.843534 (PMC9048023; doi:10.3389/fnut.2022.843534)

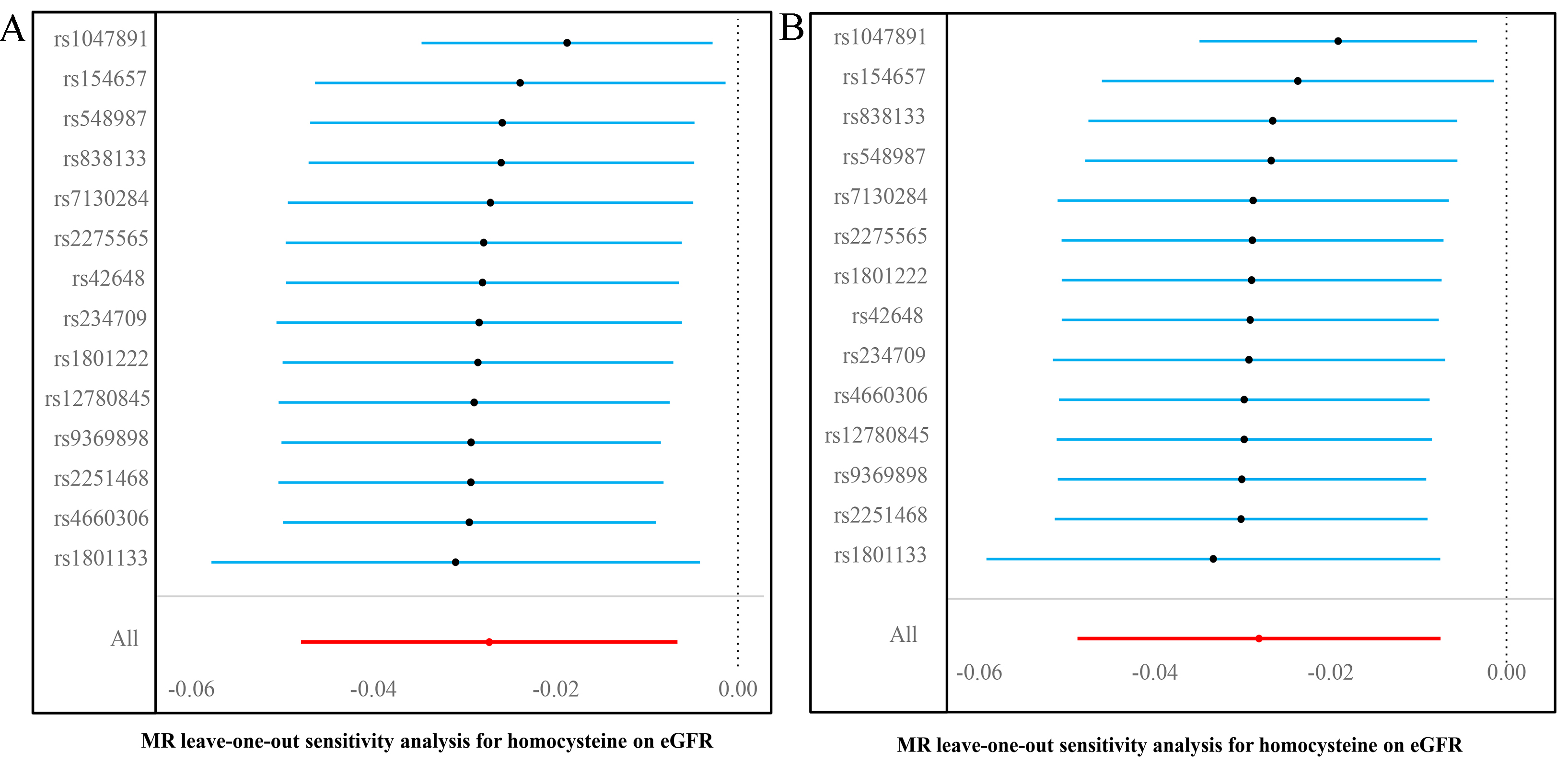

Supplement: Supplementary Figure 1 — The leave-one-out sensitivity analyses between 1-SD Hcy increase and eGFR. (A) Results of the leave-one-out analyses in the CKDGen project with diabetic patients. (B) Results of the leave-one-out analyses in the CKDGen project excluding patients with diabetes. SD, standard deviation; Hcy, homocysteine; eGFR, estimated glomerular filtration rate. [file Image_1.JPEG]

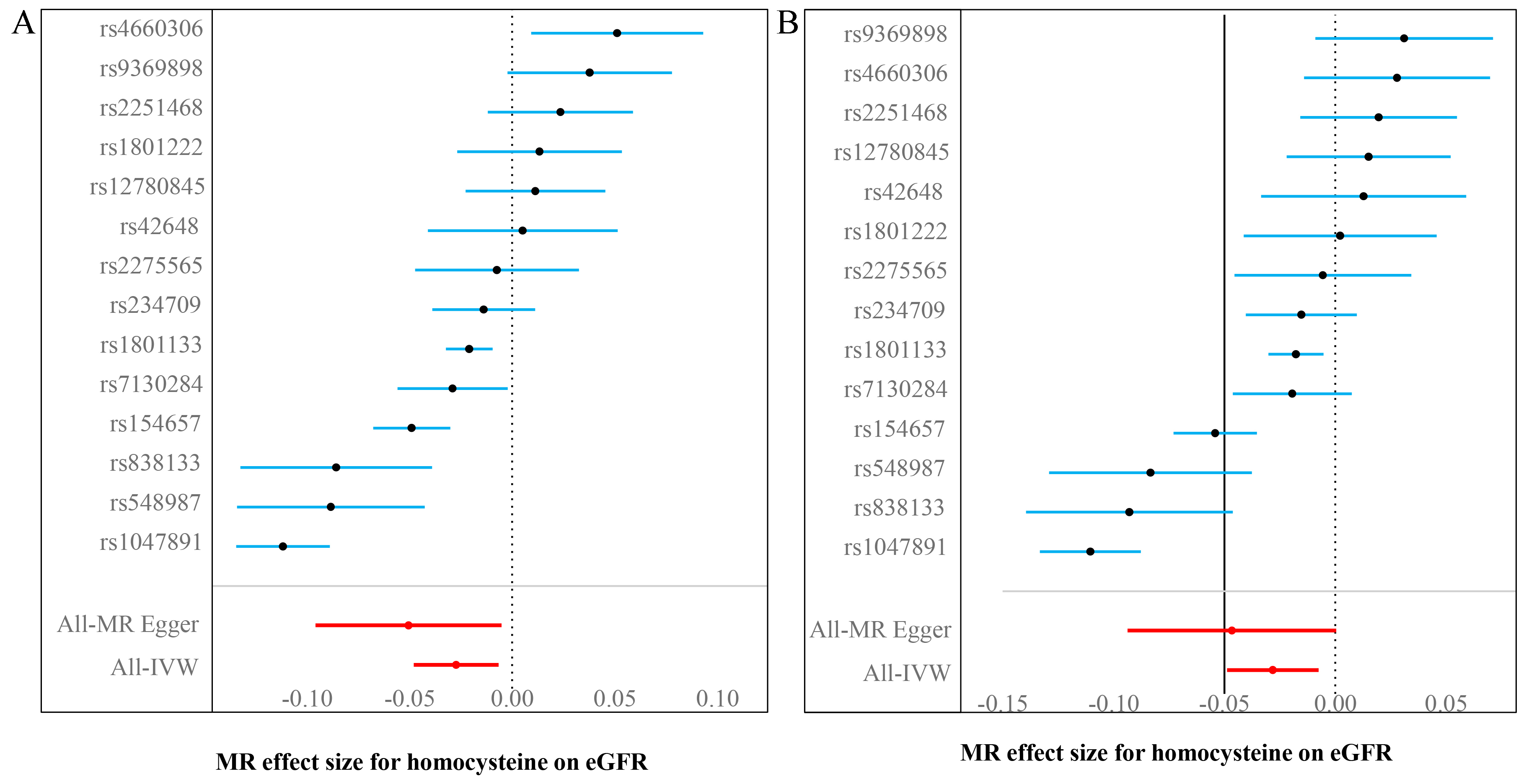

Supplement: Supplementary Figure 2 — The results of Mendelian randomization (MR) analyses of causal associations between each Hcy SNP and eGFR. (A) Forest plot of the MR analyses in the CKDGen project with diabetic patients. (B) Forest plot of the MR analyses in the CKDGen project excluding patients with diabetes. Hcy, homocysteine; eGFR, estimated glomerular filtration rate; SNP, single nucleotide polymorphism. [file Image_2.JPEG]

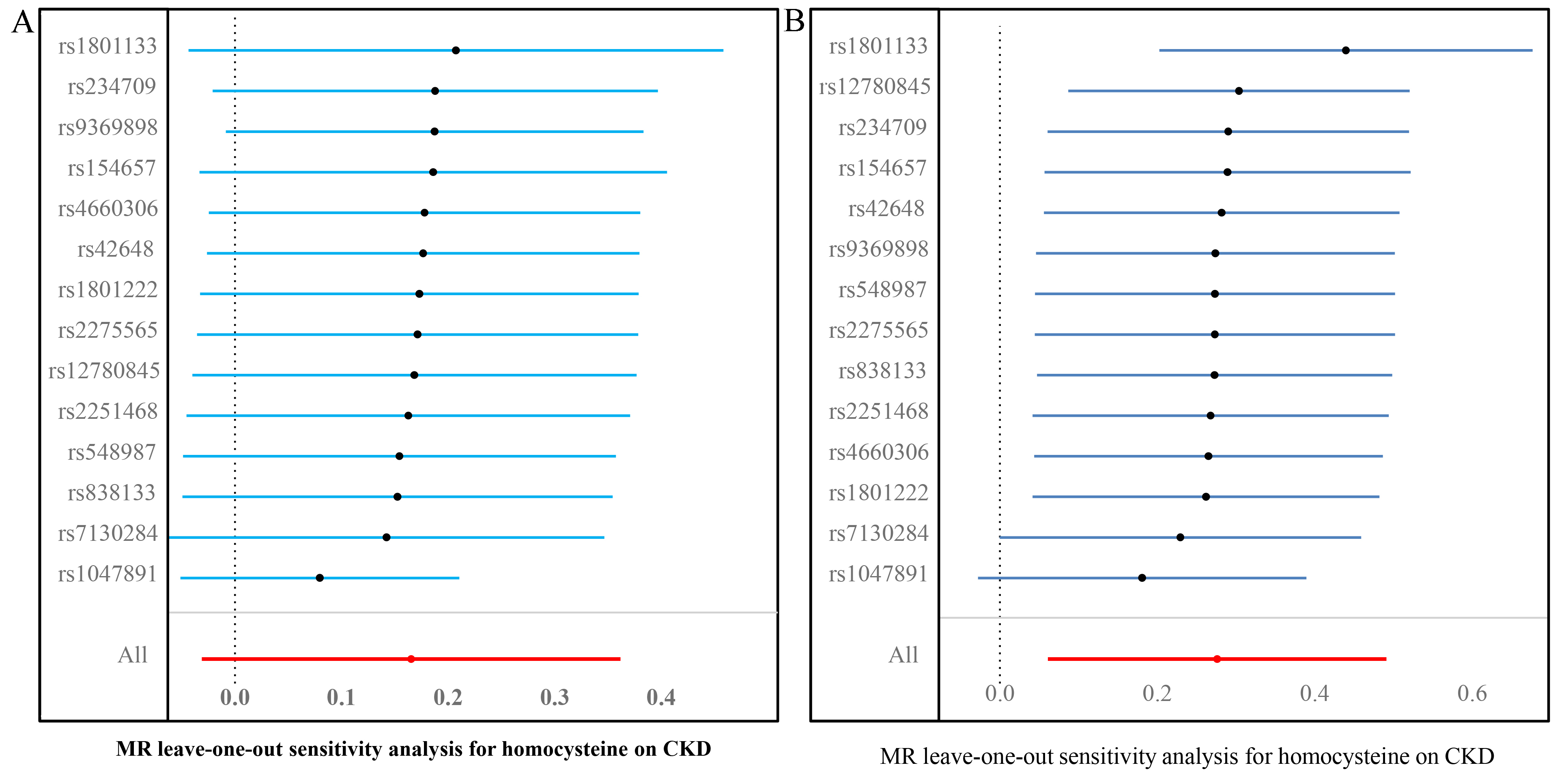

Supplement: Supplementary Figure 3 — The leave-one-out sensitivity analyses between 1-SD Hcy increase and CKD. (A) Results of the leave-one-out analyses in the CKDGen project. (B) Results of the leave-one-out analyses in the Population Architecture using Genomics and Epidemiology (PAGE) project. SD, standard deviation; Hcy, homocysteine; CKD, chronic kidney disease. [file Image_3.JPEG]

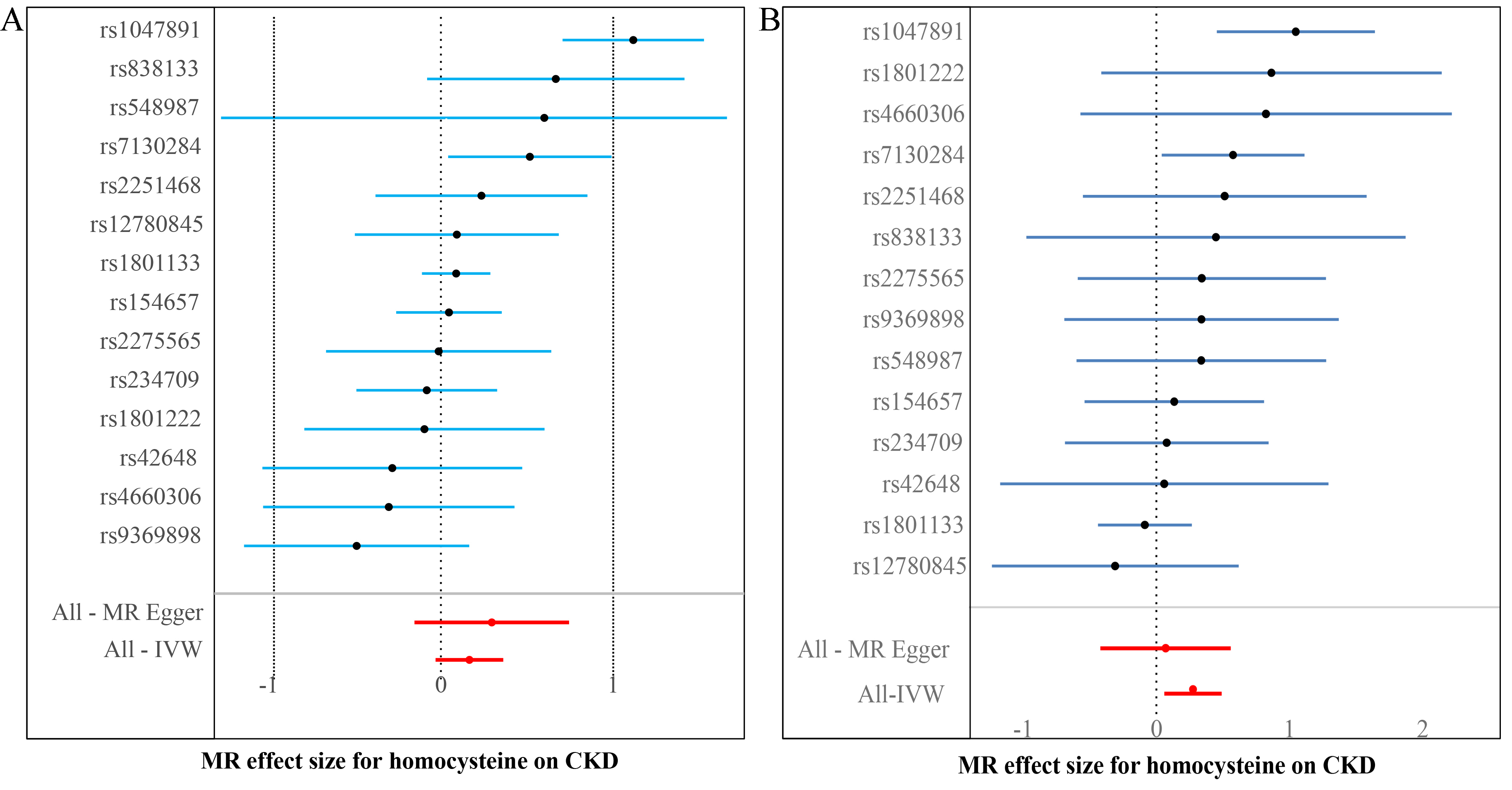

Supplement: Supplementary Figure 4 — The results of MR analyses of causal associations between each Hcy SNP and CKD. (A) Forest plot of the MR analyses in the CKDGen project. (B) Forest plot of the MR analyses in the PAGE project. Hcy, homocysteine; CKD, chronic kidney disease; SNP, single nucleotide polymorphism. [file Image_4.JPEG]

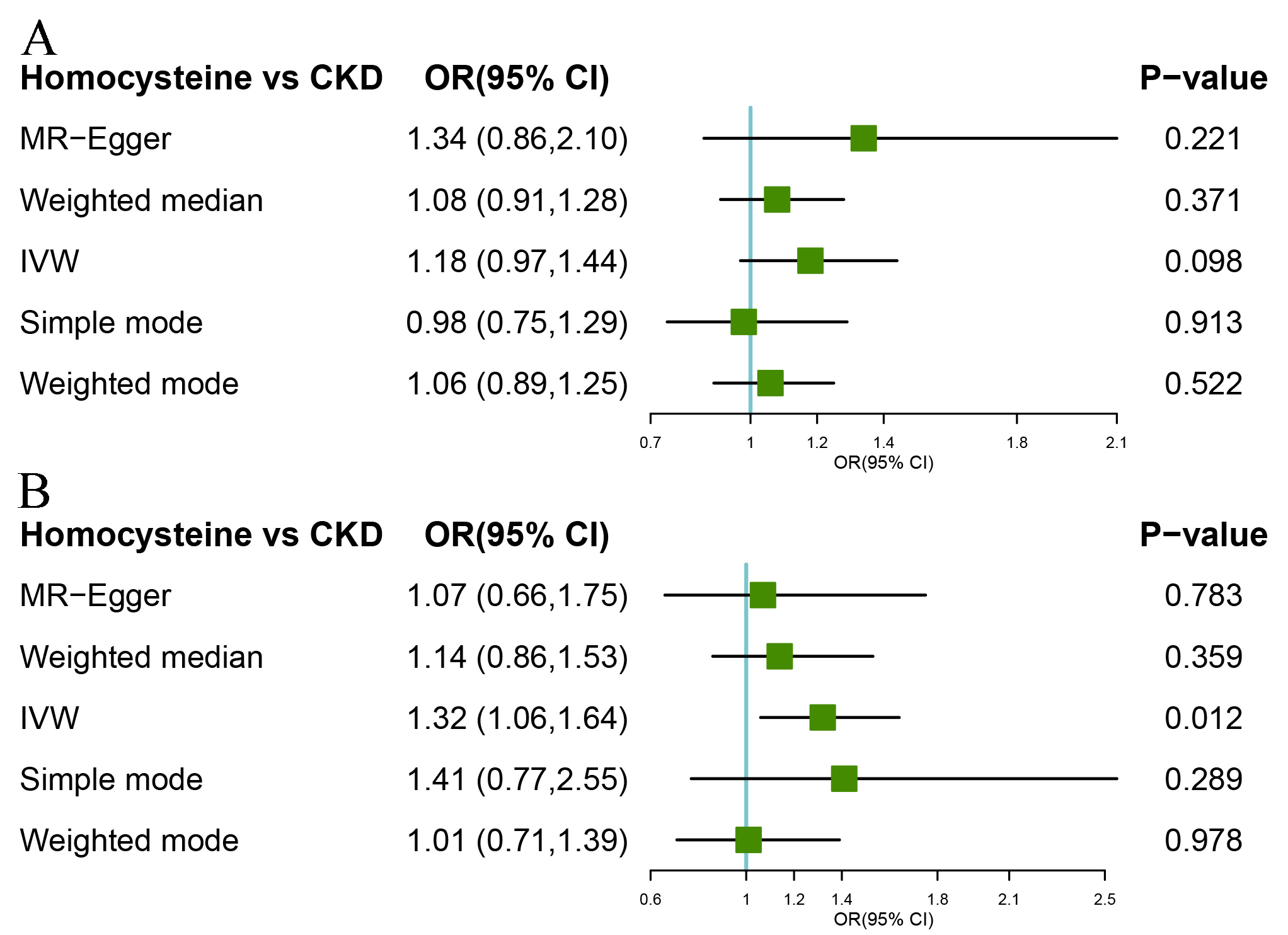

Supplement: Supplementary Figure 5 — Causal estimates of 1-SD Hcy increase on CKD. (A) Results of the MR analyses estimating the causal association between 1-SD tHcy increase and CKD in the CKDGen project. (B) Results of the MR analyses estimating the causal association between 1-SD tHcy increase and CKD in the PAGE project. SD, standard deviation; Hcy, homocysteine; IVW, inverse variance weighted method; OR, odds ratio; CKD, chronic kidney disease. [file Image_5.JPEG]
