# Supplementary material for: Genetic Evidence Supporting the Causal Role of Homocysteine in Chronic Kidney Disease: A Mendelian Randomization Study
Source: Front Nutr. 2022 Apr 14;9:843534. doi: 10.3389/fnut.2022.843534 (PMC9048023; doi:10.3389/fnut.2022.843534)
Supplement: Supplementary Table 1 — Single nucleotide polymorphisms (SNPs) used as genetic instruments in this study. SNPs, single nucleotide polymorphisms. [file Table_1.DOC]

| **Supplemental Table 1: SNPs used as genetic instruments in this study** | | | | | | | | | | |
| --- | --- | --- | --- | --- | --- | --- | --- | --- | --- | --- |
| SNP | Chr | Position | A1 | A2 | EAF | BETA | SE | P | Nearby gene | Function |
| rs1801133 | 1 | 11856378 | A | G | 0.34 | 0.158 | 0.007 | 4.30×10^-104^ | MTHFR | MTHFR encodes 5,10-methylenetetrahydrofolate reductase, which is mainly used to convert 5,10-methylenetetrahydrofolate into 5-methyltetrahydrofolate. 5-methyltetrahydrofolate can further enter the methyl transmission pathway,, providing methyl indirectly for DNA methylation and protein methylation through the methylation process of homocysteine, and consequently keep the level of homocysteine in blood at a low level. |
| rs2275565 | 1 | 237048676 | G | T | 0.79 | 0.054 | 0.009 | 2.00×10^-10^ | MTR | MTR encodes 5-methyltetrahydrofolate-homocysteine methyltransferase. This enzyme catalyzes the last step of methionine synthesis, the remethylation of homocysteine to form methionine. |
| rs1047891 | 2 | 211540507 | A | C | 0.33 | 0.086 | 0.008 | 4.60×10^-27^ | CPS1 | CPS1 encodes carbamoyl phosphate synthetase I. The protein encoded by CPS1 catalyzes the first committed step of the hepatic urea cycle. The hepatic urea cycle is responsible for the elimination of ammonia in the form of urea as well as the synthesis of arginine, a precursor of the potent vasodilatator nitric oxide. CPS1 synthesizes arbamoylphosphate from bicarbonate, ATP and ammonia using a cofactor N-acetylglutamate. Its genetic deficiency results in a rare autosomal recessive disease characterized by episodes of hyperammonemia in the neonatal period, with elevated plasma glutamine and low or absent citrulline. Nitrous oxide irreversibly inactivates the cytosolic enzyme methionine synthase by oxidizing enzyme-bound vitamin B12. Methionine synthase (MS) is a vitamin B12 dependant enzyme that catalyzes the synthesis of methionine and methyltetrahydrofolate from homocysteine and tetrahydrofolate. The inactivation of MS leads to the increase of homocysteine. |
| rs9369898 | 6 | 49382193 | A | G | 0.62 | 0.045 | 0.007 | 2.20×10^-10^ | MUT | MUT encodes for the mitochondrial enzyme methylmalonyl-Coa mutase and as such, catalyzes the isomeration of methylmalonyl-Coa into succinyl-Coa. Although its catalytic activity is hardly related to homocysteine, MUT has frequently been associated with homocysteine metabolism because it is 1 of 3 vitamin B12 dependant enzymes, along with methionine synthase and leucine aminomutase. |
| rs7130284 | 11 | 89148372 | C | T | 0.93 | 0.124 | 0.013 | 1.90×10^-20^ | NOX4 | NOX4 encodes nicotinamide-adenine dinucleotide phosphate oxidase that is highly expressed in the kidney. As such, it catalyzes the formation of the free-radical superoxide using O2 as an electron acceptor and reduced nicotinamide-adenine dinucleotide phosphate as the donor. The exact role of NOX4 in normal physiology is yet to be determined, is postulated to be associated with the regulation of renal handling of homocysteine by NOX4. |
| rs154657 | 16 | 89708096 | A | G | 0.47 | 0.096 | 0.007 | 1.70×10^-43^ | DPEP1 | DPEP1 (dipeptidase 1) is a kidney membrane enzyme that is highly expressed in the proximal convoluted tubules. It hydrolyzes a variety of dipeptides and is implicated in renal metabolism of glutathione and its conjugates, such as leukotrienes. Given DPEP1 deficiency leads to increased urinary excretion of cysteine, a precursor of homocysteine, It is hypothesized that the association between the DPEP1 and homocysteine concentration could be the result of changes in the renal handling of amino acids. |
| rs234709 | 21 | 44486964 | C | T | 0.55 | 0.072 | 0.007 | 3.90×10^-24^ | CBS | CBS encodes cystathionine-beta-synthase, which catalyzes the first step in the transsulfuration pathway of homocysteine catabolism. This enzyme irreversibly converts homocysteine to cysteine, whose catalytic action requires the participation of vitamin B6. |
| rs4660306 | 1 | 45978675 | T | C | 0.33 | 0.043 | 0.007 | 2.30×10^-09^ | MMACHC | MMACHC is the gene responsible for Cobalamin C defect of vitamin B12 metabolism. Cobalamin C defect is caused by pathogenic variants in the MMACHC gene leading to impaired conversion of dietary vitamin B12 into methylcobalamin and adenosylcobalamin. Variants in the MMACHC gene cause accumulation of methylmalonic acid and homocysteine along with decreased methionine synthesis. Patients with Cobalamin C defect mutations have both methylmalonic aciduria (MMA) and homocystinuria (HC). |
| rs548987 | 6 | 25869371 | C | G | 0.13 | 0.06 | 0.01 | 1.10×10^-08^ | SLC17A3 | The SLC17A3 gene encodes an organic anion efflux transporter that is mainly expressed in the kidney and liver. In the kidney, the encoded voltage-driven transporter excretes intracellular urate and organic anions from the blood into renal tubule cells. The association of SLC17A3 with homocysteine is widely reported but the specific mechanism remains unknown. |
| rs42648 | 7 | 89977760 | G | A | 0.6 | 0.039 | 0.007 | 2.00×10^-08^ | GTPBP10 | Small G proteins, such as GTPBP10 (GTP-binding protein 10), act as molecular switches that play crucial roles in the regulation of fundamental cellular processes such as protein synthesis, nuclear transport, membrane trafficking, and signal transduction. Currently, no study clarifies the possible mechanisms linking GTPBP10 to homocysteine. But, the decreased mitochondrial translation activity, and defects in the formation of respiratory complexes triggered by GTPBP10 knock-out may be responsible for the homocysteine. |
| rs1801222 | 10 | 17156151 | A | G | 0.34 | 0.045 | 0.007 | 8.40×10^-10^ | CUBN | The CUBN gene encodes the intrinsic factor–cobalamin receptor (cubilin), a peripheral membrane protein that acts as a receptor for intrinsic factor–vitamin B12. Vitamin B12 deficiency can lean to the increased levels of homocysteine. |
| rs2251468 | 12 | 121405126 | C | A | 0.35 | 0.051 | 0.007 | 1.30×10^-12^ | HNF1A | Hepatocyte nuclear factor 1-α (HNF1A) is found to be correlated with homocysteine with unknown mechanisms. However, HNF1A diabetes may be responsible for this. HNF1A dysfunction can impair glucose-stimulated insulin secretion. This is due to a reduced β-cell glucose uptake and metabolism resulting in low intracellular ATP levels, which, in turn, leave KATP channels open, preventing depolarization and insulin release. The disorder of glucose metabolism leads to the deficiency of folate and vitamin B12, which leads to the increase of homocysteine level in the body. |
| rs838133 | 19 | 49259529 | A | G | 0.45 | 0.042 | 0.007 | 7.50×10^-09^ | FUT2 | Absorption of B12 requires the secretion of the glycoprotein intrinsic factor (IF) from the gastric cells, binding of IF to vitamin B12 and a functional gastrointestinal absorption system2. The H-antigen synthesized by FUT2, Lewis ABO antigens, and FUT2 genotypes have all been reported to mediate H. pylori attachment to human gastric mucosa. Atrophic gastritis is a consequence of H. pylori infection and leads to reduced secretion of IF. The FUT2 secretor status has been associated with both H. pylori infection and gastritis; patients with vitamin B12 malabsorption and low levels of serum vitamin B12 have higher seroprevalence of H. pylori infection. These data suggest a potential mechanism by which vitamin B12 absorption may be reduced in carriers of the secretor genotype due to the sequelae of susceptibility to H. pylori infection compared to individuals with the nonsecretor genotype, consequently leading to the increase of homocysteine. |
| rs12780845 | 10 | 17223244 | A | G | 0.65 | 0.053 | 0.009 | 7.80×10^-10^ | TRDMT1 | TRDMT1 codes for a methyltransferase that methylates a specific RNA molecule, the aspartic acid transfer RNA (tRNAAsp). The gene is associated with increased RBC folate. In other words, the dysfunction of TRDMT1 can decrease the levels of folate and then leads to increases of homocysteine. |
